# Supplementary material for: A Framework for Intelligence and Cortical Function Based on Grid Cells in the Neocortex
Source: Front Neural Circuits. 2019 Jan 11;12:121. doi: 10.3389/fncir.2018.00121 (PMC6336927; doi:10.3389/fncir.2018.00121)
Supplement: Supplementary file 1 [file Data_Sheet_1.pdf]

Supplementary Material

A Framework for Intelligence and Cortical Function Based on Grid Cells in the Neocortex

Jeff Hawkins[1*], Marcus Lewis[1], Mirko Klukas[1], Scott Purdy[1], Subutai Ahmad[1]

[1]Numenta, Inc., Redwood City, CA, USA

*** Correspondence:**
jhawkins@numenta.com

1 Displacement Cells

Here we describe a possible network mechanism for a layer of displacement cell modules, derived from a layer of grid cell modules. We first illustrate the mechanism for a single module, and then describe multiple modules.

In the lower box of **Figure 1A**, we show the cells in a single grid cell module. In this simple example there are 9 cells, arranged in a 3X3 lattice. For convenience we assign (x,y) coordinates to each cell where x and y can be 0, 1, or 2. The cells in this lattice are arranged such that neighboring cells represent neighboring positions. For example, if cell $G_{1,1}$ is active for a location on an object, a movement one step to the right will cause $G_{2,1}$ to become active. Since the cells tile space, an additional movement one step to the right will cause $G_{0,1}$ to become active.

Each grid cell module is paired with a displacement module (upper box of **Figure 1A**). Each cell in the displacement module corresponds to a particular relative movement. It becomes active if any of the grid cell pairs corresponding to its displacement become active in the appropriate order. For example, cell $D_{1,0}$, representing a displacement one step to the right, would become active if grid cell $G_{2,2}$ becomes active after grid cell $G_{1,2}$. It would also become active if cell $G_{1,1}$ becomes active after $G_{0,1}$, etc. A possible neural implementation is that these pairs of grid cells connect to independent dendritic segments on cell $D_{1,0}$ such that any of these transitions cause $D_{1,0}$ to become active.

A grid cell module can be thought of as implementing a residue number system (Omondi and Premkumar, 2007; Sreenivasan and Fiete, 2011). Viewed in this light a displacement module implements residual subtraction operations. If the active grid cell moves from location $G_{x1,y1}$ to $G_{x2,y2}$, the displacement cell in position $((x2 - x1)\ modulo\ 3, (y2 - y1)\ modulo\ 3)$ will become active. Each cell in the displacement module connects to 9 pairs of grid cells. Cell $D_{1,0}$ thus becomes active for any of the following 9 pairs of cells:

$G_{0,2} \rightarrow G_{1,2}$	$G_{1,2} \rightarrow G_{2,2}$	$G_{2,2} \rightarrow G_{0,2}$
$G_{0,1} \rightarrow G_{1,1}$	$G_{1,1} \rightarrow G_{2,1}$	$G_{2,1} \rightarrow G_{0,1}$
$G_{0,0} \rightarrow G_{1,0}$	$G_{1,0} \rightarrow G_{2,0}$	$G_{2,0} \rightarrow G_{0,0}$

Figure 1B shows all the transitions within the grid cell module that cause $D_{1,0}$ to become active. These transitions form the input to the displacement cells.

The number of possible displacements is tied to the number of cells in a grid cell module. Note that the magnitude and direction of a shift is ambiguous within a module. You cannot distinguish a displacement two steps to the right from a displacement one step to the left. However, the activity across displacement modules will be unique to a particular directional displacement in the same way that the activity across grid cell modules is unique to a location.

Now let us see how this single module network represents an example composite object such as the logo on the coffee mug. **Figure 1C** shows two points on the composite object. Each point has a location in the space of the coffee mug as well as a location in the space of the logo. In our example, suppose that attending to point P_1 on the coffee mug invokes grid cell $G_{0,1}$ in the coffee mug space, and attending to it on the logo invokes cell $G_{1,1}$ in the logo space. The activity of these two cells will invoke displacement cell $D_{1,0}$. This displacement cell will remain active, regardless of where you move on the composite object. Suppose, you switch to point P_2 and this is one step below P_1. Grid cells $G_{0,0}$ and $G_{1,0}$ will become active in the coffee mug and logo spaces, respectively. However, the relative positions of these two cells will still invoke displacement cell $D_{1,0}$.

Due to the modulo operation of displacement cells it is not possible to uniquely distinguish a composite object with just one module. In our simple example, there is a 1 in 9 chance that another composite object will invoke the same displacement cell. However, with a collection of independent grid cell modules and associated displacement modules, the combined activity is highly likely to be unique. If there are M modules with N cells in each module, the chance that two composite objects will invoke an identical set of displacement cells is 1 in N^M. Note that as you sense different locations on the composite object, the active grid cells will change, but the activity in the displacement cells will be stable. Thus, the overall pattern in the displacement layer is unique to a particular pair of objects, in a specific relative configuration.

This is a provisional file, not the final typeset article

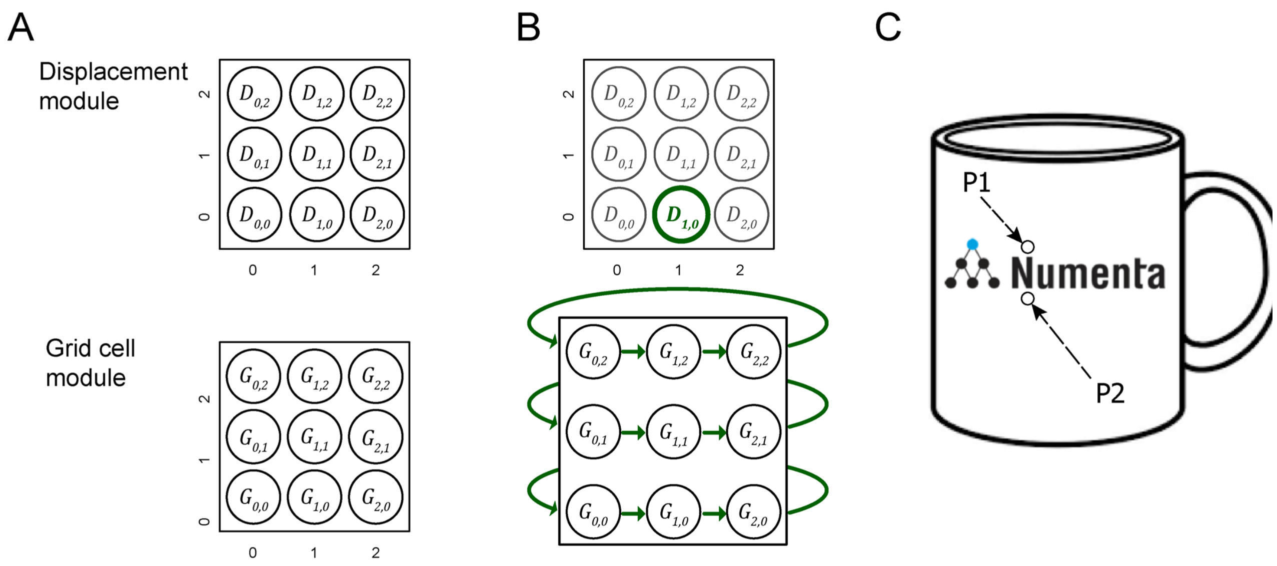

Figure 1. **(A)** Cells in a single grid cell module (lower square) and its corresponding displacement module (upper square). In this example each module contains 9 cells, arranged in a 3X3 lattice with associated (x,y) coordinates. Cells are arranged such that neighboring cells represent neighboring positions or displacements. For convenience we show the same number of cells in both modules. **(B)** Shows the transitions that cause displacement cell $D_{1,0}$(green) to become active. Any of the 9 transitions (green arrows) will cause the cell to become active. **(C)** Two points on a coffee cup with an embedded logo. Attending to P_1 in the coffee cup space followed by attending to P_1 in the logo space will activate two different grid cells and a single displacement cell. If you attend to P_2, in cup space and then logo space, the active grid cells will be different than with P_1 , but the active displacement cell will not change.

A displacement layer as described above can encode relative shifts in position. In general, to completely specify a composite object, it is also necessary to encode relative scaling, rotation, and perhaps other transformations. In the coffee cup example the size and angle of the logo is just as important as the positioning. We would notice a discrepancy if the logo were half the size or tilted at 45 degrees. Residue number systems are powerful enough to handle many numerical operations. It is therefore possible that these other transformations can also be represented by operations on grid cell modules. This is an area of ongoing research for us.

2 References

Omondi, A., and Premkumar, B. (2007). *Residue Number Systems: Theory and Implementation*. London: Imperial College Press.

Sreenivasan, S., and Fiete, I. (2011). Grid cells generate an analog error-correcting code for singularly precise neural computation. *Nat. Neurosci.* 14, 1330–1337. doi:10.1038/nn.2901.
